# Stability of the CpG island methylator phenotype during glioma progression and identification of methylated loci in secondary glioblastomas

**DOI:** 10.1186/1471-2407-14-506

**Published:** 2014-07-10

**Authors:** Victoria K Hill, Thoraia Shinawi, Christopher J Ricketts, Dietmar Krex, Gabriele Schackert, Julien Bauer, Wenbin Wei, Garth Cruickshank, Eamonn R Maher, Farida Latif

**Affiliations:** 1 Centre for Rare Diseases and Personalised Medicine and Department of Medical & Molecular Genetics, School of Clinical and Experimental Medicine, University of Birmingham College of Medical and Dental Sciences, Edgbaston, Birmingham, UK; 2 Department of Neurosurgery, University Hospital Carl Gustav Carus Dresden, Technical University of Dresden, Dresden, Germany; 3 Department of Pathology, University of Cambridge, Tennis Court Road, Cambridge CB2 1QP, UK; 4 School of Cancer Sciences, University of Birmingham, Birmingham, UK; 5 Department of Neurosurgery, University of Birmingham and Queen Elizabeth Hospital Birmingham, Birmingham, UK

**Keywords:** Primary and secondary glioblastoma (pGBM, sGBM), HumanMethylation450, Methylation, IDH1, CIMP

## Abstract

**Background:**

Grade IV glioblastomas exist in two forms, primary (*de novo*) glioblastomas (pGBM) that arise without precursor lesions, and the less common secondary glioblastomas (sGBM) which develop from earlier lower grade lesions. Genetic heterogeneity between pGBM and sGBM has been documented as have differences in the methylation of individual genes. A hypermethylator phenotype in grade IV GBMs is now well documented however there has been little comparison between global methylation profiles of pGBM and sGBM samples or of methylation profiles between paired early and late sGBM samples.

**Methods:**

We performed genome-wide methylation profiling of 20 matched pairs of early and late gliomas using the Infinium HumanMethylation450 BeadChips to assess methylation at >485,000 cytosine positions within the human genome.

**Results:**

Clustering of our data demonstrated a frequent hypermethylator phenotype that associated with *IDH1* mutation in sGBM tumors. In 80% of cases, the hypermethylator status was retained in both the early and late tumor of the same patient, indicating limited alterations to genome-wide methylation during progression and that the CIMP phenotype is an early event. Analysis of hypermethylated loci identified 218 genes frequently methylated across grade II, III and IV tumors indicating a possible role in sGBM tumorigenesis. Comparison of our sGBM data with TCGA pGBM data indicate that *IDH1* mutated GBM samples have very similar hypermethylator phenotypes, however the methylation profiles of the majority of samples with WT *IDH1* that do not demonstrate a hypermethylator phenotype cluster separately from sGBM samples, indicating underlying differences in methylation profiles. We also identified 180 genes that were methylated only in sGBM. Further analysis of these genes may lead to a better understanding of the pathology of sGBM vs pGBM.

**Conclusion:**

This is the first study to have documented genome-wide methylation changes within paired early/late astrocytic gliomas on such a large CpG probe set, revealing a number of genes that maybe relevant to secondary gliomagenesis.

## Background

Gliomas are classified into 4 grades according to the WHO classification system. These range from curable World Health Organization (WHO) grade I tumors (pilocytic astrocytomas) to the highly malignant WHO grade IV glioblastoma (GBM) with mean survival < 1year. In between these two grades are WHO grade III malignant tumors (anaplastic astrocytomas) with median survival rates of 2–3 years after diagnosis and WHO grade II (diffuse astrocytomas) considered as low grade gliomas with median survival rates of 6–8 years after diagnosis [1,2]. Glioblastomas are subdivided into 2 distinct types, primary grade IV glioblastoma (pGBM or *de novo* glioblastomas) that account for >90% of the cases, usually affecting older patients and develop rapidly after a short clinical history and without evidence of a less malignant precursor lesion. While secondary glioblastomas (sGBM) develop slowly through progression from lower grade diffuse or anaplastic astrocytomas and more commonly occur in younger patients. pGBM and sGBM represent not only clinically distinct entities but also demonstrate distinct genetic heterogeneity. For example, pGBM demonstrate mutation of the *PTEN* gene and frequent loss of heterozygosity on chromosome 10q (inclusive of the *PTEN* gene locus), amplification of *EGFR*, deletions of *CDKN2A (p16)*, while sGBM and their lower grade precursor lesions have frequent mutations of the *TP53* gene and the *IDH1* gene [3-7]. Recent studies have also looked at genetic alterations in early and late paired secondary samples [8].

In recent years large scale genome-wide epigenetic studies have been performed with the aim of developing clinically relevant biomarkers for glioblastoma [9-11]. A good example is the epigenetic silencing of the *MGMT* promoter that has provided an exciting and clinically relevant epigenetic marker in gliomas. The *MGMT* gene encodes for an *O*-6-methylguanine methyltransferase that removes alkyl groups from the O-6 position of guanine. Thus loss of its activity greatly impairs a cells ability to tolerate alkylating agents and studies have shown that *MGMT*-promoter methylation is associated with longer survival of patients treated with alkylating agents such as temozolomide [12,13]. Recently, the Cancer Genome Atlas (TCGA) research network identified a CpG island methylator phenotype (CIMP) in a subset of human gliomas with distinct clinical and molecular features, including improved survival outcomes for those gliomas demonstrating CIMP [10]. The gain of function mutations within the isocitrate dehydrogenase 1 gene (*IDH1*) are thought to be largely responsible for the glioma hypermethylator phenotype due to the massively increased production of the 2-hydroxyglutarate oncometabolite and have recently been shown to be sufficient to result in a hypermethylator phenotype in glioma cell lines [14,15]. At least some individual genes have demonstrated differential methylation frequencies in grade IV pGBM and sGBM samples [16] and although much progress has been made in assessing genome-wide methylation of pGBM tumors, much less is known about genome-wide methylation in early grade tumors and their subsequent higher grade sGBM manifestations.

Recent technological advances have made it possible to quantitatively assess genome-wide methylation at the individual CpG loci level using the Illumina Infinium BeadChips. The most recent version of this BeadChip (Infinium HumanMethylation450 BeadChip) is able to quantitatively assess the levels of methylation at specific CpG loci throughout the genome, including CpG islands and regions of much lower CpG dinucleotide density. In this report we utilized these comprehensive Infinium HumanMethylation450 BeadChip arrays to define genome-wide methylation in paired samples of early/late astrocytic gliomas and to demonstrate any alterations induced by progression.

## Methods

### DNA samples

Forty DNA samples from 20 astrocytoma/glioma patients were used in this study. These patient samples consisted of; 10 WHO grade II astrocytomas, 15 WHO grade III astrocytomas and 15 WHO grade IV glioblastomas. The 40 DNA samples represent 20 cases of paired early and late lesions from the same patient. The DNA was extracted from tissue samples consisting of a minimum of 80% tumor. The DNA from four non-disease brain samples was used to provide the normal, expected levels of methylation. Ethical guidelines were followed for patient sample collection and all samples have been anonymised. Research was conducted according to the principles expressed in the Declaration of Helsinki. Patients gave written informed consent for analysis of tumor samples. The study was approved by the Institutional Ethics Committees of University of Technology Dresden and University of Birmingham.

### Illumina array

The Illumina Infinium HumanMethylation450 array (Illumina, San Diego, CA, USA) was performed on 0.5 μg bisulfite modified patient DNA according to manufacturers’ instructions. Bisulfite modification of DNA and array hybridization was carried out by Cambridge Genomics Services. Raw data was obtained using Genome Studio software from Illumina. The raw data were processed using the lumi R [17] package to correct for the color bias present due to the use of different dye on the array. To correct this bias, Infinium type I and type II are separated, then both channel are also separated and the color bias is corrected using a within array smooth quantile normalization. After correction the two channels and probe types are combined and a between array quantile normalization is performed. The beta score are then calculated. The raw files have been deposited in NCBI’s Gene Expression Omnibus [18] and are accessible through GEO Series accession number GSE58298.

Probes demonstrating detection p-values greater than 0.01 in any sample were removed along with probes located on the X and Y chromosomes. To ensure tumor specific hypermethylation, probes showing a beta value ≥0.25 in any of the four normal samples were also removed. Hypermethylation was subsequently determined as a beta value ≥0.5. This was considered relevant if present in >30% tumor samples. Additional filtering was achieved limiting selection to genes for which the hypermethylation criteria were met in ≥3 probes associated to that gene.

### Clustering

The top 2000 most variable loci for each clustering event were determined by selecting the 2000 probes with the greatest standard deviation across all the given samples. Clustering was performed using the Cluster3 program (http://bonsai.hgc.jp/~mdehoon/software/cluster/software.htm#ctv) and visualized using the Java TreeView program (http://jtreeview.sourceforge.net/). Unsupervised hierarchical clustering was performed using the Euclidean based algorithm.

### Clone sequencing

Clone sequencing was used for array validation. 0.5 μg of DNA for each sample was bisulfite modified using the Qiagen EpiTect kit (Qiagen, Heidelberg, Germany) according to manufacturers’ instructions. PCR reactions were performed using FastStart Taq DNA polymerase (Roche, West Sussex, UK) on a semi-nested basis for all genes using the primers listed in Additional file 1. A touchdown PCR program for primary and secondary reactions using gene specific annealing temperatures was performed. Selected PCR products were cloned into the pGEM-T easy vector (Promega, Madison, WI, USA) according to manufacturers’ instructions and cultured overnight at 37°C. Up to 12 colonies were selected for single colony PCR using primer sequences F: 5′- TAATACGACTCACTATAGGG -3′ and R: 5′- ACACTATAGAATACTCAAGC -3′. PCR products were cleaned for sequencing using thermosensitive alkaline phosphatase (Fermentas UK, York, UK) and Exonuclease I (NEB, Ipswich, MA, USA) and then sequenced using cycle sequencing on an ABI 3730 (Applied Biosystems, Carlsbad, CA, USA). Methylation indexes were calculated as a percentage of the number of methylated CpGs out of the total number of CpGs sequenced.

### *IDH1* and *IDH2* mutation status

Previously described primers were used to amplify 129 bp and 150 bp fragments of the *IDH1* and *IDH2* genes [19]. The *IDH1* forward primer 5′-CTCCTGATGAGAAGAGGGTTG-3′ and *IDH1* reverse primer 5′-TGGAAATTTCTGGGCCATG-3′ were used to sequence codon 132 and the *IDH2* forward primer 5′-TGGAACTATCCGGAACATCC-3′ and *IDH2* reverse primer 5′-AGTCTGTGGCCTTGTACTGC-3 were used to sequence codon 172 of *IDH2*. Twenty nanograms of genomic DNA were used as starting material for a 25 μl total volume PCR reaction using Go Taq polymerase. An annealing temperature of 58°C was used for 35 cycles. PCR products were bi-directionally sequenced using cycle sequencing on an ABI 3730x (Applied Biosystems, Carlsbad, CA, USA).

### TCGA samples

Illumina Infinium HumanMethylation450 BeadChip array data was used for the following 19 TCGA primary glioblastomas: TCGA-06-5416, TCGA-06-0171, TCGA-26-5136, TCGA-06-0190, TCGA-06-5418, TCGA-06-0210, TCGA-26-5135, TCGA-26-5134, TCGA-26-5132, TCGA-12-5295, TCGA-06-5414, TCGA-06-0211, TCGA-26-5133, TCGA-06-5417, TCGA-06-0221, TCGA-26-1442, TCGA-06-6389, TCGA-06-6701, TCGA-15-1444. All array data was downloaded from the TCGA Data Portal (https://tcga-data.nci.nih.gov/tcga/tcgaHome2.jsp). *IDH1* and *IDH2* mutation status for these tumors was identified using the cBioPortal for Cancer Genomics (http://www.cbioportal.org/public-portal/).

## Results

To determine whether aberrant DNA methylation differs between early and late secondary glioma lesions we have used the new Illumina Infinium HumanMethylation450 BeadChip array on 40 astrocytic secondary glioma tumors, consisting of 20 pairs of early and late lesions for individual patients and four normal brain samples. Of the 20 patient paired samples; 5 pairs are WHO grade II astrocytomas progressing to grade III astrocytomas, 5 pairs are WHO grade II astrocytomas progressing to WHO grade IV glioblastomas, and 10 pairs are grade III astrocytomas progressing to grade IV glioblastomas. In order to adjust for potential bias based on the differences in probe design between Illumina Type I/II probes we ran all raw data through a correction pipeline prior to analysis. In addition, these samples had been assessed for *IDH1* and *IDH2* mutation status, 14 out of 20 (70%) samples demonstrated mutation in the IDH1 R132 codon. No *IDH2* mutations were detected (Additional file 2: Table S1).

### CIMP is an early event in secondary gliomagenesis that can be retained throughout progression

**Figure 1:**
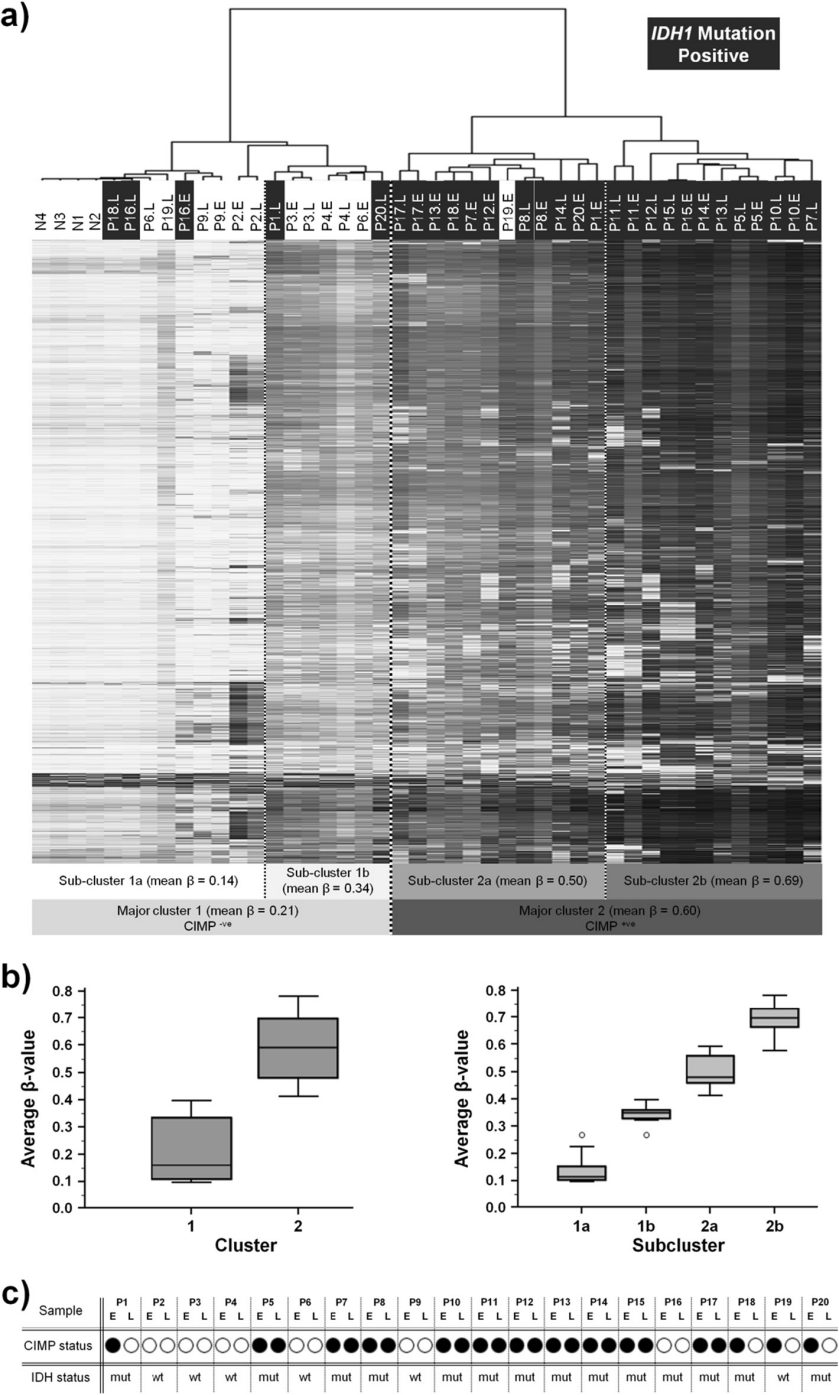
**Clustering analysis. 1a**. Hierarchical euclidean based clustering of the 2000 most variable loci. Samples split into 2 major cluster groups designated as being either CIMP^+ve^ or CIMP^-ve^ with each major cluster splitting into 2 sub-groups. Normal samples clustered together and are labeled N1-N4, tumor samples are labeled with their pair number (P#) followed by either E or L to denote early or late lesion respectively. **1b**. Box and whisker plots of cluster group ANOVAs. **1c**. CIMP status as determined by clustering is shown with a black or white circle representing CIMP^+ve^ and CIMP^-ve^ respectively. *IDH1* mutation status is shown as either mutant (mut) or wild-type (wt) for the p.R132H change.

Unsupervised clustering of the 2000 most variable loci in all 40 samples plus normal controls produces two major clusters: major cluster 1 (n = 20 samples; mean beta value = 0.21) and major cluster 2 (n = 24 samples; mean beta value = 0.60) (p < 0.001; ANOVA) (Figure 1a, b). Each major cluster can be further sub-divided into 2 sub-clusters: sub-clusters 1a and 1b (n = 13 and n = 7 samples respectively; mean beta values 0.14 and 0.34 respectively) and sub-clusters 2a and 2b (n = 12 samples in each cluster; mean beta values 0.50 and 0.69 respectively) (p < 0.001; ANOVA). Mean beta values for samples within each sub-cluster differ significantly in all comparisons (p < 0.05; ANOVA) (Figure 1b). Samples within major cluster 2 demonstrate a high level of methylation throughout the most variable 2000 loci indicating the CpG island methylator phenotype (CIMP) and these samples were designated CIMP^+ve^ with all but one sample (P19E) demonstrating an *IDH1* mutation (Figure 1). Within our most variable 2000 loci were probes for genes previously associated with a CIMP phenotype in GBM [10]. Samples in major cluster 1 appear to be negative for the CIMP phenotype and were designated CIMP^–ve^ with sub-cluster 1a appearing to be notably normal-like, including all the control normal samples, whilst sub-cluster 1b has low level methylation. Interestingly, major cluster 1 included several *IDH1* mutation positive samples as well as all the IDH1 mutation negative samples except for P19E (Figure 1a). In general, *IDH1* mutation negative samples (P2, P3, P4 and P9) demonstrated very similar methylation patterns between early and late grades (Figure 1a). For all but one (P16) of the *IDH1* positive samples, the lower grade sample demonstrated distinct CIMP and this is suggestive that it is a very early event in secondary gliomagenesis. In addition, no sample gained CIMP during progression suggesting that it occurs early on or not at all. In progression to the later grades the *IDH1* positive sample split into two categories; those samples that retain a very similar methylation profile after progression (P5, P8, P10, P11, P15, P17) and those that demonstrate a partially remaining CIMP^+ve^ between early and late lesions or greatly reduced (becoming CIMP^-ve^) degree of methylation after progression (P1, P7, P12, P13, P14, P18, P20) (Figure 1a). Thus, in total, progression through to higher grades had little effect on the genome-wide methylation for 10 of the 20 pairs (50%) and no effect on CIMP status for 16 of the 20 pairs (80%). The P19 sample acted as though an IDH mutation was present and that it fell into the second category of *IDH1* mutation positive samples. Although the sample was negative for *IDH1* or *IDH2* mutation it could possibly have another mutation capable of causing a similar effect, such as a *TET2* mutation, that was not assessed for. The *IDH1* positive P16 sample acted more like an *IDH1* negative sample for unknown reasons and was retained for further analysis.

### Identification of hypermethylated loci dependent upon glioma grade

**Figure 2:**
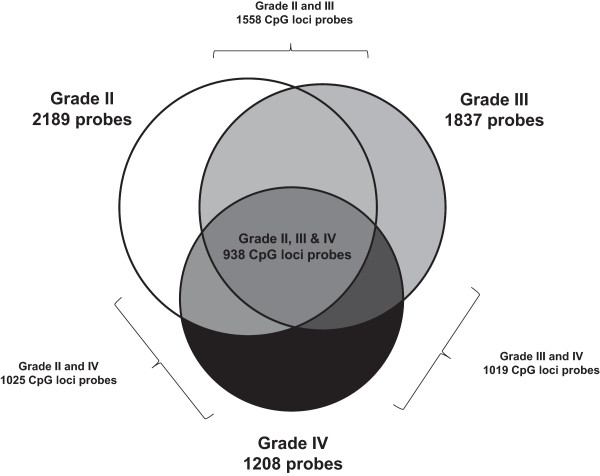
**The cross-over between hypermethylated CpG loci probes in grade II, III and IV samples is illustrated with a venn diagram.** Numbers refer to the number of hypermethylated probes that belong to genes present in the list by ≥3 probes.

To initially discern a list of differentially methylated loci between normal and tumor samples we first split the samples into grade II, III and grade IV groups and identified hypermethylated loci within each group. Following removal of all probes showing a β-value ≥0.25 in any of the four normal samples, the remaining probes were considered hypermethylated if >30% of tumor samples showed a β-value of ≥0.5. When using these criteria: 6024 CpG loci were identified as being hypermethylated in grade II astrocytomas of which 4374 were associated with a gene; 5295 CpG loci were identified as being hypermethylated in grade III astrocytomas of which 3772 were associated with a gene; 3329 CpG loci were identified as being hypermethylated in grade IV glioblastomas of which 2397 were associated with a gene. This trend of decreasing methylation levels is in agreement with our clustering data above and with previous studies [11,20]. Further analysis was carried out only with probes that were associated with genes. The location of differentially methylated loci with respect to gene features was very similar for each grade, the majority being within the gene body (34.9%, 34.9% and 36.1% for grades II, III and IV respectively) and within 1500 bp of the transcription start site (22.6%, 21.7% and 20.2% for grades II, III and IV respectively), this largely followed the distribution of analyzed CpG probes as determined by array design (Additional file 3: Figure S1). However, we saw a very different distribution of hypermethylated CpG loci compared to design array when assessing the genomic location. In this case, the majority of hypermethylated probes fell within CpG islands (67.9%, 72.7% and 73.8% for grade II, III and IV respectively) while only 35.6% of total analyzed probes fell within these regions. In contrast, we saw very few hypermethylation events within open sea locations (7.1%, 7.1% and 5.5% for grades II, III and IV, respectively) compared to the total number of CpG loci analyzed within these locations (31.4%) (Additional file 3: Figure S1). Due to the large number of probes per gene in the Infinium HumanMethylation450 BeadChip array we were able to further refine our gene lists by removing genes that had limited CpG hypermethyaltion events. Removal of genes that were not represented by ≥3 probes resulted in 2189 relevant hypermethylated probes representing 496 genes in astrocytoma grade II samples, 1837 relevant hypermethylated probes representing 427 gene in astrocytoma grade III samples and 1208 relevant hypermethylated probes representing 279 genes in grade IV glioblastomas. Of the 2189 loci that are hypermethylated in grade II astrocytomas, approximately 24.9% (n = 544) are specifically hypermethylated within this group, in contrast, grade III and IV samples showed a lower level of specific grade methylation (10.8%, n = 198 and 8.4%, n = 102) respectively (Figure 2).

### Identification of the hypermethylated loci conserved during tumor grade progression

To try and identify genes important throughout secondary gliomagenesis it was assumed that genes hypermethylated in all glioma grades would be the most relevant. This analysis identified 939 hypermethylated CpG loci across all grades for further analysis. This list represents 232 genes and was, as before, reduced to 218 genes (represented by 914 CpG loci) by selecting genes that were represented in the list by ≥3 CpG loci probes. The gene list and beta values for these probes are provided in Additional file 4: Tables S2 and S3 respectively. Three genes (*ALS2CL*, *GNMT* and *WNK2*) were chosen from the list of 218 genes to confirm array values with regard to methylation. We chose two genes that had not previously been shown to be methylated in GBM (*ALS2CL* and *GNMT*) and one gene that has (*WNK2*) [21] for this technical validation of array results. Results from clone sequencing confirmed β-values >0.5 are representative of methylation and that very low β-values correspond to no methylation (Additional file 5: Figure S2; Additional file 1). Use of the Ingenuity Pathway Analysis software identified 47.7% (104/218) of genes as falling within five molecular and cellular function groups; cell morphology, cellular movement, cellular development, cellular growth and proliferation, and cellular assembly and organization. Of these 104 genes, 39 have been previously associated with cancer (Additional file 6: Table S4-S5).

### Identification of sGBM preferentially methylated targets

**Figure 3:**
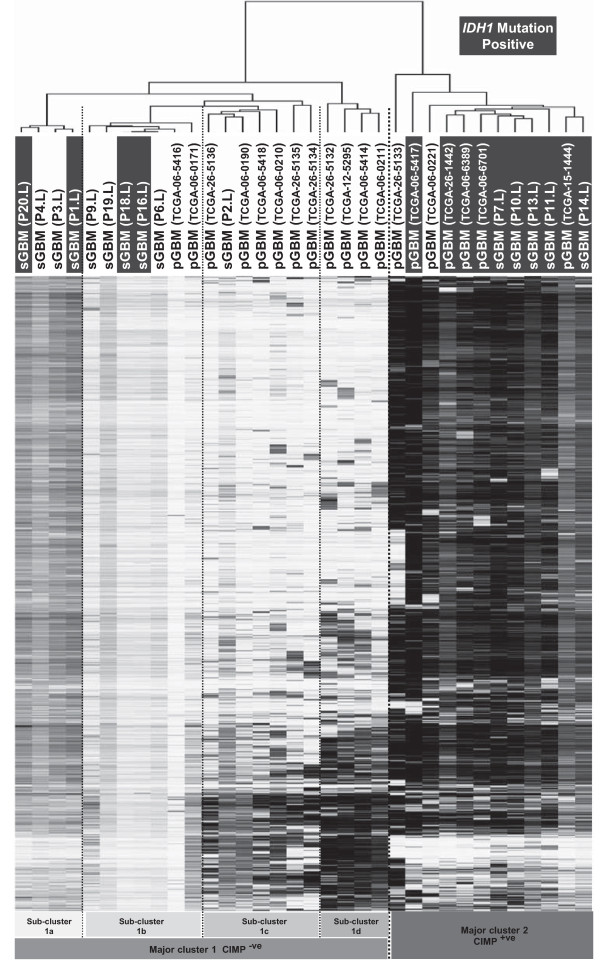
**Hierarchical Euclidean based clustering of the 2000 most variable loci.** Samples split into 2 major cluster groups containing either CIMP^+ve^ or CIMP^-ve^ samples (major cluster 2 and 1 respectively). Major cluster 1 split into four sub-clusters. *IDH1* mutated samples are highlighted with a black box. sGBM samples are labeled with their pair number (P#) followed by L to denote late lesion. pGBM samples are labeled with respective TCGA sample names.

Since there is evidence for primary and secondary gliomas having different genetic attributes and this is one of the first examples of the Illumina Infinium HumanMethylation450 BeadChip arrays on secondary gliomas we used a subset of the publically available Infinium HumanMethylation450 BeadChip primary GBM TCGA datasets to compare methylation in primary and secondary grade IV glioblastomas to determine any global methylation differences. To avoid any bias due to our sGBM data being adjusted for Illumina Type I/II probes (see Methods), we chose to use our sGBM data prior to adjustment for this particular analysis. Using 15 of TCGA grade IV pGBM and our 15 grade IV sGBM data, we clustered the most variable 2000 loci and observed that the pGBM samples largely clustered together, while the sGBM samples clustered into two separate groups dependent upon their CIMP phenotype (data not shown). This suggests there is also epigenetic heterogeneity between pGBM and sGBM, at least in terms of DNA methylation, but interestingly there were three of the pGBM samples that clustered within the sGBM CIMP^+ve^ group, one of which had the *IDH1* p.R132H change. To further expand this analysis we then downloaded all pGBM *IDH1* p.R132H mutated samples that had available Infinium HumanMethylation450 BeadChip methylation data (4 additional samples, there were no *IDH2* mutated samples). Clustering of the total 19 pGBM samples with our 15 grade IV sGBM samples showed the CIMP phenotype within all five pGBM *IDH1* mutated samples, which clustered together with our sGBM CIMP^+ve^*IDH1* mutation positive samples, indicating the CIMP^+ve^ phenotype induced by *IDH1* mutation in pGBM is similar in sGBM. Two additional pGBM samples also clustered within this group, completing the smaller of the two major cluster groups (Figure 3). Of the larger, CIMP^-ve^ cluster, samples split into four sub-clusters, dependent predominantly on pGBM/sGBM status. Sub-clusters 1c and 1d contained all but two pGBM samples and only one sGBM sample whilst the remaining two sub-clusters contain all but one sGBM CIMP^-ve^ samples (Figure 3), indicating methylated targets of CIMP^-ve^ primary and secondary grade IV glioblastomas differ significantly. Three of the four sGBM CIMP^-ve^*IDH1* mutation positive samples are the samples that exhibited CIMP in the earlier lesion but not the later lesion, whilst the fourth sGBM and its paired earlier lesion were CIMP^-ve^. Comparison of methylated gene lists for sGBM and pGBM samples (irrelevant of CIMP status) identified 180 genes that were only methylated in sGBM samples according to our criteria (Additional file 7: Table S6). We also identified 338 genes that were only methylated in pGBM samples (Additional file 7: Table S6) and 123 genes methylated in both. Reassuringly, only two genes were present in the pGBM specific list and our earlier list of 218 genes that were methylated across grade II, III and IV secondary gliomas. Of the 180 genes that were sGBM specific from this analysis, 115 were present in our list of 218 genes across grade II, III and IV secondary gliomas (Additional file 8: Figure S3). This discrepancy is most probably due to a combination of looking only at grade IV samples and using data unadjusted for Type I/II Illumina probes. Ingenuity analysis identified a substantial number of genes associated with cancer in both lists, with substantially more in the pGBM only list; 20% and 54% of genes within the sGBM and pGBM lists respectively. Considerable differences were observed between the molecular and cellular functions of genes within each list (Table 1). The pGBM gene list is enriched for genes that alter or control gene expression which in turn may affect cellular development and growth and proliferation. In contrast, the sGBM only list is enriched for genes that affect cell death, survival and maintenance pathways that would need to be altered or abrogated for tumorigenesis. Thus, the differing patterns of methylation between these two subtypes of glioma may provide differing advantages to these tumor cells.

**Table 1:** Ingenuity Pathway Analysis software assessment of molecular and cellular functions of exclusively methylated genes in either pGBM grade IV glioblastomas or sGBM grade IV glioblastomas

**sGBM Ingenuity analysis**^**(w)**^	**Category**^**(x)**^	**No. of Genes**^**(y)**^	**p-value range**^**(z)**^
Diseases and disorder	Cancer	36/180 (20.0%)	2.52E-04 – 1.05E-02
Molecular and cellular functions	Cellular compromise	12/180 (6.7%)	3.53E-06 – 8.01E-03
Molecular and cellular functions	Cellular assembly and organization	44/180 (24.4%)	1.56E-05 – 7.76E-03
Molecular and cellular functions	Cell morphology	45/180 (25.0%)	1.29E-04 – 1.29E-04
Molecular and cellular functions	cell death and survival	47/180 (26.1%)	2.52E-04 – 1.02E-02
Molecular and cellular functions	Cellular function and maintenance	38/180 (21.1%)	2.52E-04 – 1.05E-02
**pGBM Ingenuity analysis**^**(w)**^	**Category**^**(x)**^	**No. of Genes**^**(y)**^	**p-value range**^**(z)**^
Diseases and disorder	Cancer	183/338 (54.1%)	7.67E-09 – 2.68E-04
Molecular and cellular functions	Gene expression	104/338 (30.8%)	7.23E-23 – 1.28E-04
Molecular and cellular functions	Cellular development	124/338 (36.7%)	4.44E-20 – 7.94E-04
Molecular and cellular functions	Cell morphology	73/338 (21.6%)	1.16E-09 – 8.32E-04
Molecular and cellular functions	Cellular movement	90/338 (26.6%)	4.41E-09 – 6.52E-04
Molecular and cellular functions	Cellular growth and proliferation	116/338 (34.3%)	2.45E-08 – 7.69E-04

Table 1 shows the top diseases and disorders and 5 molecular and cellular functions of genes exclusively methylated in sGBM samples (top) and pGBM (bottom) samples. For each table: (w) type of ingenuity analysis used (diseases and disorders or molecular and cellular functions); (x) category of either the disease/disorder or molecular/cellular function; (y) the total number of genes within the list per category out of the total number of genes expressed, also shown as a percentage; (z) p-value range of this number of genes within the list falling into each category.

## Discussion

Secondary GBM represents a smaller subset (5%) of GBM tumors which develop from preexisting lower grade tumors (grade II/III), are more often seen in younger patients and patients with sGBM have longer survival times [3]. These tumors demonstrate distinct genetic heterogeneity compared to primary GBM, including a considerably greater mutation rate of the *IDH1* gene that has been shown to result in a CpG island methylator phenotype (CIMP). In this report we have used the latest Illumina Infinium HumanMethylation450 BeadChips to assess the genome-wide methylation of 20 secondary glioblastomas and their matching lower grade precursors. Sandoval et al. [22] recently validated the Illumina Infinium HumanMethylation450 BeadChip array and demonstrated that this latest array consistently and significantly detects CpG methylation changes in the HCT-116 colorectal tumor cell line in comparison with normal colon mucosa or HCT-116 cells with defective DNA methytransferases [22]. While whole-genome bisulfite sequencing is the gold standard for comprehensive mapping of methylation events, it is still expensive and requires a high level of specialization. However, the Illumina Infinium HumanMethylation450 BeadChip offers a powerful technique for better understanding of the DNA methylation changes occurring in human diseases at a reasonable cost. Our study represents the first to utilize the Illumina Infinium HumanMethylation450 BeadChips to evaluate epigenetic changes occurring during glioma progression.

We demonstrated that these samples had the expected high levels of *IDH1* mutation and that in the lower grade precursors this nearly uniformly resulted in a CIMP phenotype. We saw one case (P16, early and late lesions) where there was evidence of an *IDH1* mutation but no CIMP phenotype. We also saw one case (P19.E) where there was no evidence of *IDH1* or *IDH2* mutation but was CIMP positive. However, it has previously been suggested that even when negative for the known *IDH1* p.R132H mutation, it is possible that other *IDH1* mutations could be present in some cases that might therefore potentially affect CIMP status [23]. The early presentation of *IDH1* mutation and CIMP that we have seen in our study suggests this is an early and important event in gliomagenesis and that if not acquired at an early stage is not gained during progression as no later stage glioblastoma presented with CIMP where the precursor did not. Although the total number of samples is small the large degree of *IDH1* mutation and CIMP argues strongly that this is true. In addition to increased overall survival, *IDH1* mutation status has been shown to correlate with genetic features including the presence of *MGMT* methylation and codeletion of 1p and 19q, as well as inversely correlating with *EGFR* amplification, chromosome 10 loss and chromosome 7 polysomy [5,24] and therefore if we had been able to analyze a larger sample set, it would have been interesting to look at the relationship between these factors.

The effects of tumor grade progression on the genome-wide methylation of these paired samples of sGBM tumors and their earlier lower grade lesions could be assessed in the most comprehensive manner to date due to the large amount of data provided by the Illumina Infinium HumanMethylation450 BeadChips. Firstly, as mentioned above samples lacking CIMP in their precursor lesions never gained it via progression, presumably due to the early gain of some other genetic or environmental factor capable of driving gliomagenesis without the subsequent need for hypermethylation. While those samples presenting with CIMP in their precursor lesion, largely in association with *IDH1* mutation, split approximately in half to follow two paths after progression. Some samples appeared to fully retain and maintain CIMP in their higher grade lesions whatever level CIMP hypermethylation was observed within the lower grade precursor lesions, presumably due to the importance of this high level of general hypermethylation to the tumors survival. Interestingly, some samples notably reduced their levels of general hypermethylation, some retaining what we defined as CIMP and some losing it. This could potentially be due the initial lower grade lesion demonstrating epigenetic heterogeneity with different cells having differing hypermethylation patterns that together present as CIMP positive. If a subset of these cells contained hypermethylation of a particular tumor suppressor that resulted in a considerable growth advantage then these cells could grow out and progress to be the higher grade lesion. This lesion would still have the evolutionary pressure to maintain the hypermethylation of this specific tumor suppressor but not necessarily the need to maintain a global methylation phenotype, although in general you would expect some degree of maintenance by the *IDH1* mutation, it is plausible that due to changing tumor heterogeneity this would be visualized at a lesser extent. Unfortunately we were unable to assess different regions from within the same tumor to investigate this hypothesis. Furthermore, we observed that these differences were not simply due to pairs progressing from grade II to grade III compared to grade III to grade IV or grade II to grade IV. Due to the relatively small size of our cohort we were unable to identify the specific genetic differences that may support this hypothesis as we would assume them to be tumor specific. Nonetheless this is an interesting observation that could possibly affect the effectiveness of therapies based on demethylating agents on these tumors. Naturally, we would assume they would be more effective in samples that at some stage demonstrated CIMP but they may still be effective in samples that do not demonstrate CIMP in the later grades if CIMP was present in the precursor lesion. It is hard to estimate whether a demethylating agent would be more effective on tumors dependent on global hypermethylation or are reliant on the hypermethylation of only a small number of targets. Promisingly, 5-azacitidine has recently been shown to be effective in reducing selected promoter methylation, tumor growth, cell proliferation and inducing differentiation in an *in vivo* primary xenograft *IDH1* mutant glioma [25].

Further evidence for the loss of some hypermethylation due to tumor grade progression was observed when the levels of hypermethylated loci and genes were assessed simply by the grade of each tumor rather than looking for differences between paired samples. We noticed a trend towards decreasing levels of methylated targets with increasing tumor grade which has previously been documented [11,20]. This loss of methylation as tumors progress to later grades may indicate changes in tumor heterogeneity resulting in refinement of the most beneficial effects of hypermethylation as proposed above, but could also represent a potential increase in normal contamination as the tumor becomes more invasive and thus the tumor sample more intermingled with normal.

By analyzing grade II, III and IV tumors separately, we were able to identify a list of genes where hypermethylation was retained in all 3 grades, likely representing the most generally important methylated genes within this cohort of sGBM tumors. This identified preferential hypermethylation of several genes associated with cell morphology, cellular movement, cellular development, cellular growth and proliferation, and cellular assembly and organization, with many of these select genes having been previously associated with cancer. Due to the relatively small number of tumors assessed, this analysis would greatly benefit from expansion into a larger cohort that could highlight which genes and pathways are most important to sGBM gliomagenesis and progression.

By comparison of methylation profile of our grade IV lesions with a subset of the publically available methylation profiles of grade IV pGBM provided by the Cancer Genome Atlas (TCGA) network we demonstrated that in general the methylation profiles between these two tumor types differ in a similar manner to their respective genetic alterations. This was further observed when comparing the functions of genes commonly hypermethylated in grade IV sGBMs compared to grade IV pGBMs with sGBMs preferentially hypermethylating genes involved in cell death, survival and maintenance pathways and pGBMs preferentially hypermethylating genes that alter or control gene expression. Interestingly, a small number of the pGBM tumors demonstrated CIMP that was also largely associated with *IDH1* mutation, demonstrating a very similar hypermethylation profile to CIMP positive grade IV sGBM. This represented a specific epigenetic overlap between a subset of the pGBM and sGBM tumors. Included in this were two pGBM tumors exhibiting CIMP that lacked mutation in *IDH1* or *IDH2* that could possibly retain other mutations capable of resulting in CIMP such as could be present in our 19^th^ pair. Overall, this small sGBM/pGBM analysis offers an insight into different tumorigenic processes giving rise to these different types of GBM tumors.

## Conclusions

In summary, this data offers an insight into different epigenetic, methylation-related processes that give rise to these different types of GBM tumors and provides interesting rationales for further study of this kind on much larger cohorts. The increased use of genome-wide analysis of methylation using technologies such as the Illumina Infinium HumanMethylation450 BeadChips, that are relatively cheap and can be performed using both archival tissue DNA from FFPE blocks and small amounts of DNA acquired from biopsies, may well increase their usefulness as diagnostic or therapeutic markers. Thus, providing a greater understanding on these tumor specific methylation patterns may prove useful in a number of ways.

## Competing interests

The authors declare that they have no competing interests.

## Authors’ contributions

FL and VKH designed the study and drafted the manuscript. ERM and GC helped design the study. VKH carried out the clone sequencing and in silico/bioinformatics analysis. TS carried out IDH1 mutation analysis. CJR carried out in silico/bioinformatics analysis. DK and GS provided the DNA samples and clinical information. JB developed the correction pipeline. WW helped with bioinformatic analysis. All authors’ read and approved the final manuscript.

## Pre-publication history

The pre-publication history for this paper can be accessed here:

http://www.biomedcentral.com/1471-2407/14/506/prepub

## Supplementary Material

Additional file 1 **Primer sequences are provided for *ALS2CL***, ***GNMT *and *WNK2 *beta value validation analysis.** Click here for file

Additional file 2: Table S1 Sample information is provided for the patient samples used in this study. (a) Pair numbers used in this study. (b) sample numbers used in this study. (c) WHO grade of each tumour given as either astrocytoma (astro) grade II or III; or GBM (glioblatoma multiforme); astrocytoma WHO grade IV. (d) KPS; Karnofsky Performance Score at the time of initial admission. (e) tumor location (hemisphere) (f) RTx; whether radiotherapy was received (g) CTx; whether chemotherapy was received (h) IDH1 mutation status; mutated (mut) or wild type (wt) for the recurrent p.R132H mutation. Click here for file

Additional file 3: Figure S1 Pie charts for each grade illustrate the distribution of hypermethyaled CpG loci with respect to gene features or genomic location. Gene features include CpG loci within the following regions: 1^st^ exon, 3′UTR, 5′UTR, gene body, within 1500 base pairs of the transcription start site (TSS1500) or within 200 bp of the transcription start site (TSS200). Genomic locations include: CpG islands (island), north CpG island shelves (N shelf), south CpG island shelves (S shelf), north CpG island shores (N shore) south CpG island shores (S shore) or unclassified regions (open sea). Hypermethylated CpG loci distributions are almost identical in each grade. We also show the distribution of CpG islands that were analyzed for hypermethylation events with respect to gene feature or genomic location, as determined by the array design. These include all probes on the array that associated with a gene, were not on either the X or Y chromosome and were not associated with a SNP. Click here for file

Additional file 4: Table S2 This table contains the gene symbols for probes methylated in grade II, III and IV samples in 3 or more probes. **Table S3.** This table contains probe beta values for the genes listed in **Table S2.** Click here for file

Additional file 5: Figure S2 Clone sequencing results are shown for CpG island regions of three genes; ALS2CL, WNK2 and GNMT. Black and white circles represent methylated and unmethylated CpG dinucleotides respectively and each line represents a single clone. Methylation indexes are given for each sample as a percentage of methylated CpGs out of the total number of CpGs analysed. CpG dinucleotides analysed by the infinium assay are indicated by an arrow and beta values for these loci are shown next to each sample. Click here for file

Additional file 6 **Ingenuity pathway analysis results for the 218 genes hypermethylated across grade II, III and IV tumor samples. Table S4.** shows the top 5 molecular and cellular functions alongside the number of genes falling within each category and the p-value range. Gene symbols are given for genes that fall within any of these 5 categories. Gene symbols in bold indicate genes that have previously been associated with cancer. **Table S5.** illustrates the top 3 gene networks of the 218 hypermethylated genes and the genes present within each of these networks. Click here for file

Additional file 7: Table S6 Gene lists for hypermethylated genes exclusively in sGBM or pGBM samples, following comparison between the lists for the two tumor types. Click here for file

Additional file 8: Figure S3 Venn diagram to illustrate the cross-over between hypermethylated genes within the grade IV pGBM and grade IV sGBM specific lists in addition to the original list of universally methylated genes across grade II, III and IV secondary glioma samples. Click here for file
