# Supplementary material for: Stability of the CpG island methylator phenotype during glioma progression and identification of methylated loci in secondary glioblastomas
Source: BMC Cancer. 2014 Jul 10;14:506. doi: 10.1186/1471-2407-14-506 (PMC4227105; doi:10.1186/1471-2407-14-506)
Supplement: Additional file 3: Figure S1 — Pie charts for each grade illustrate the distribution of hypermethyaled CpG loci with respect to gene features or genomic location. Gene features include CpG loci within the following regions: 1st exon, 3′UTR, 5′UTR, gene body, within 1500 base pairs of the transcription start site (TSS1500) or within 200 bp of the transcription start site (TSS200). Genomic locations include: CpG islands (island), north CpG island shelves (N shelf), south CpG island shelves (S shelf), north CpG island shores (N shore) south CpG island shores (S shore) or unclassified regions (open sea). Hypermethylated CpG loci distributions are almost identical in each grade. We also show the distribution of CpG islands that were analyzed for hypermethylation events with respect to gene feature or genomic location, as determined by the array design. These include all probes on the array that associated with a gene, were not on either the X or Y chromosome and were not associated with a SNP. [file 1471-2407-14-506-S3.pptx]

## Slide 1
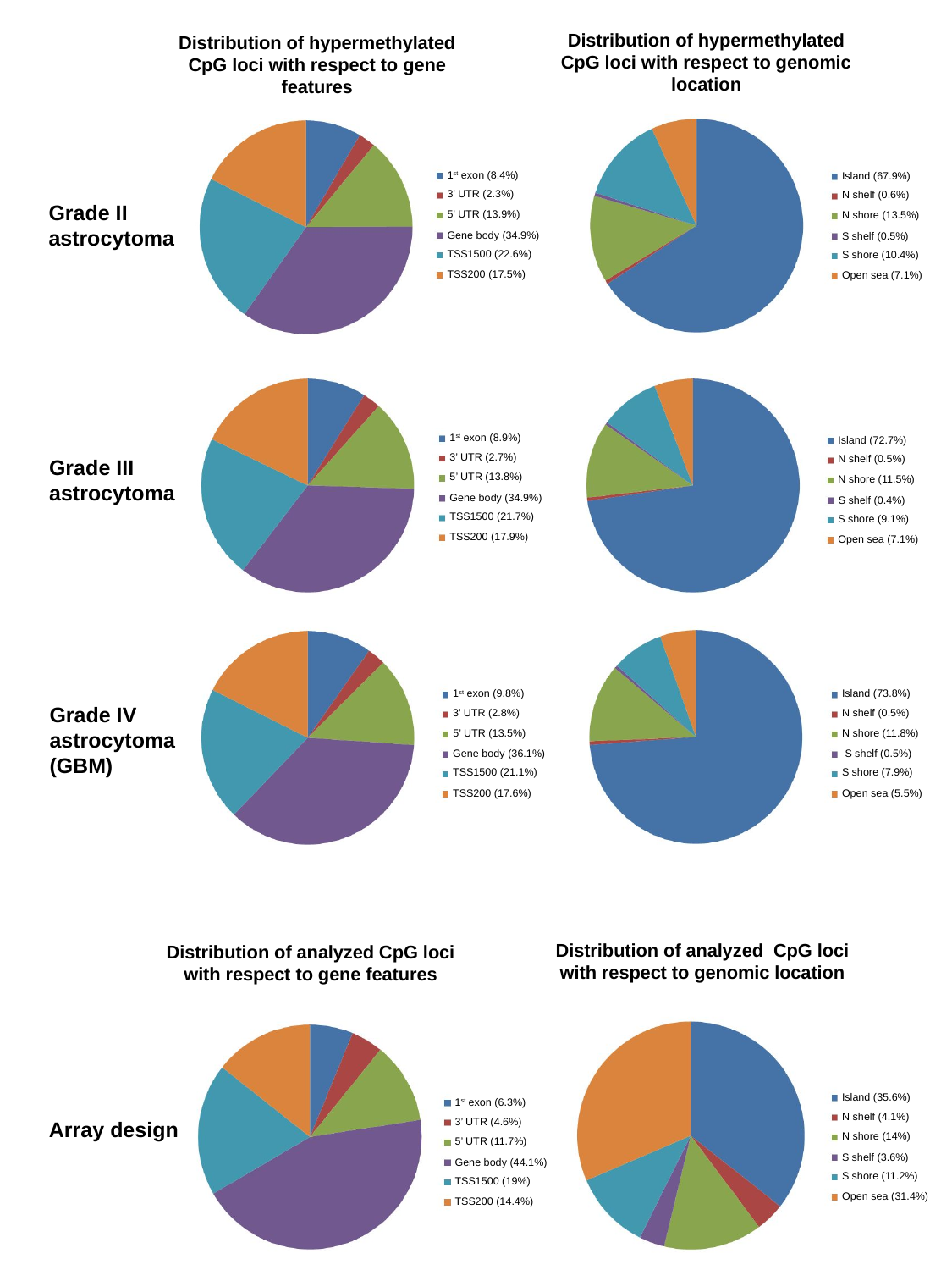

Distribution of hypermethylated CpG loci with respect to genomic location
Distribution of hypermethylated CpG loci with respect to gene features
Island (67.9%)
N shelf (0.6%)
N shore (13.5%)
S shelf (0.5%)
S shore (10.4%)
Open sea (7.1%)
1st exon (8.4%)
3’ UTR (2.3%)
5’ UTR (13.9%)
Gene body (34.9%)
TSS1500 (22.6%)
TSS200 (17.5%)
Grade II astrocytoma
1st exon (8.9%)
3’ UTR (2.7%)
5’ UTR (13.8%)
Gene body (34.9%)
TSS1500 (21.7%)
TSS200 (17.9%)
Island (72.7%)
N shelf (0.5%)
N shore (11.5%)
 S shelf (0.4%)
S shore (9.1%)
Open sea (7.1%)
Grade III astrocytoma
1st exon (9.8%)
3’ UTR (2.8%)
5’ UTR (13.5%)
Gene body (36.1%)
TSS1500 (21.1%)
TSS200 (17.6%)
Island (73.8%)
N shelf (0.5%)
N shore (11.8%)
 S shelf (0.5%)
S shore (7.9%)
Open sea (5.5%)
Grade IV astrocytoma (GBM)
Distribution of analyzed CpG loci with respect to genomic location
Distribution of analyzed CpG loci with respect to gene features
Island (35.6%)
N shelf (4.1%)
N shore (14%)
S shelf (3.6%)
S shore (11.2%)
Open sea (31.4%)
1st exon (6.3%)
3’ UTR (4.6%)
5’ UTR (11.7%)
Gene body (44.1%)
TSS1500 (19%)
TSS200 (14.4%)
Array design
