# Supplementary material for: Stability of the CpG island methylator phenotype during glioma progression and identification of methylated loci in secondary glioblastomas
Source: BMC Cancer. 2014 Jul 10;14:506. doi: 10.1186/1471-2407-14-506 (PMC4227105; doi:10.1186/1471-2407-14-506)
Supplement: Additional file 5: Figure S2 — Clone sequencing results are shown for CpG island regions of three genes; ALS2CL, WNK2 and GNMT. Black and white circles represent methylated and unmethylated CpG dinucleotides respectively and each line represents a single clone. Methylation indexes are given for each sample as a percentage of methylated CpGs out of the total number of CpGs analysed. CpG dinucleotides analysed by the infinium assay are indicated by an arrow and beta values for these loci are shown next to each sample. [file 1471-2407-14-506-S5.pptx]

## Slide 1
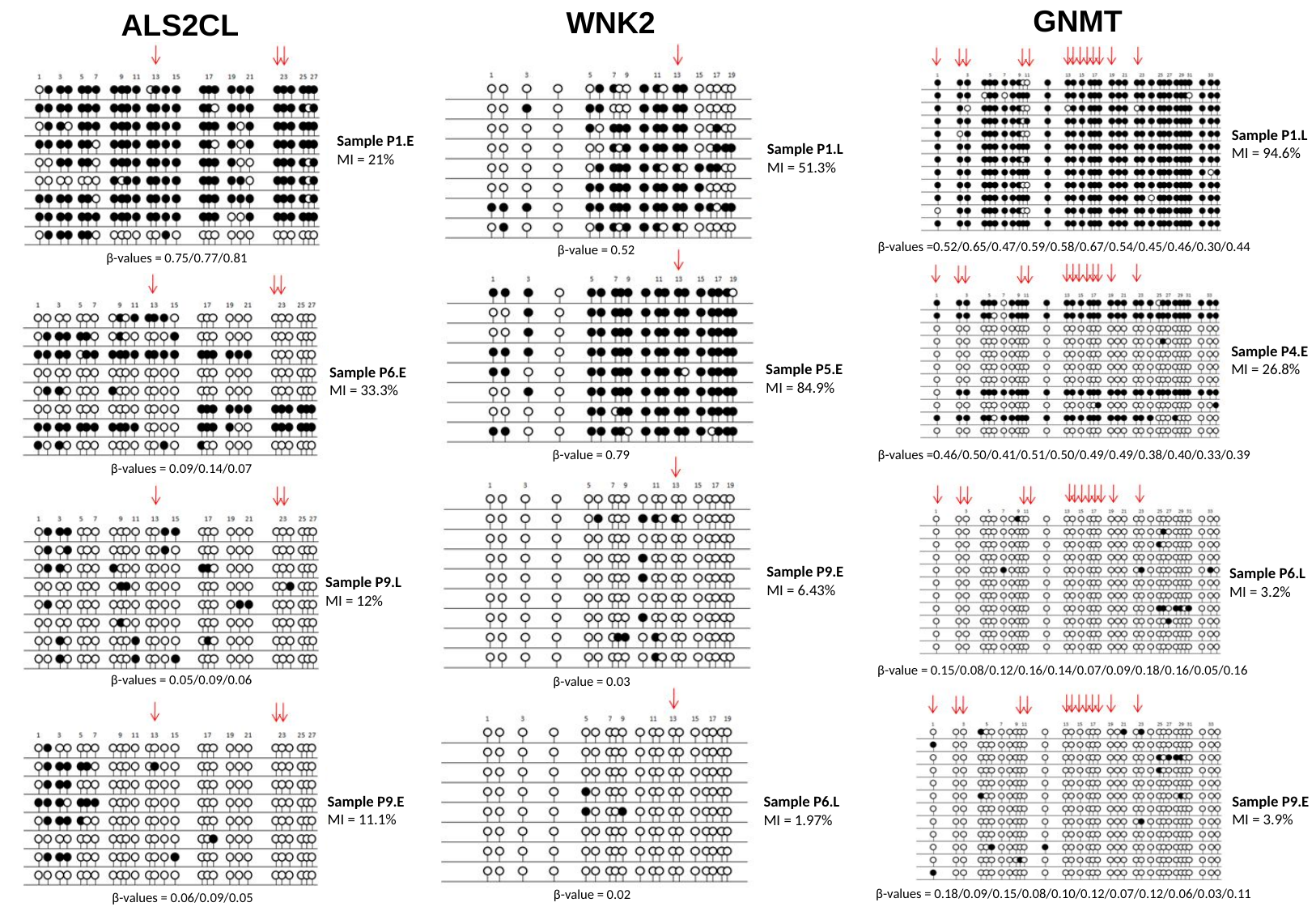

ALS2CL
Sample P1.E
MI = 21%
β-values = 0.75/0.77/0.81
Sample P6.E
MI = 33.3%
β-values = 0.50/0.51/0.55
β-values = 0.09/0.14/0.07
Sample P9.L
MI = 12%
β-values = 0.05/0.09/0.06
Sample P9.E
MI = 11.1%
β-values = 0.06/0.09/0.05
GNMT
Sample P1.L
MI = 94.6%
Sample P4.E
MI = 26.8%
β-values =0.46/0.50/0.41/0.51/0.50/0.49/0.49/0.38/0.40/0.33/0.39
Sample P6.L
MI = 3.2%
β-value = 0.15/0.08/0.12/0.16/0.14/0.07/0.09/0.18/0.16/0.05/0.16
Sample P9.E
MI = 3.9%
β-values = 0.18/0.09/0.15/0.08/0.10/0.12/0.07/0.12/0.06/0.03/0.11
WNK2
Sample P1.L
MI = 51.3%
β-value = 0.52
Sample P5.E
MI = 84.9%
β-value = 0.79
Sample P9.E
MI = 6.43%
β-value = 0.03
Sample P6.L
MI = 1.97%
β-value = 0.02
β-values =0.52/0.65/0.47/0.59/0.58/0.67/0.54/0.45/0.46/0.30/0.44
