# Supplementary material for: Stability of the CpG island methylator phenotype during glioma progression and identification of methylated loci in secondary glioblastomas
Source: BMC Cancer. 2014 Jul 10;14:506. doi: 10.1186/1471-2407-14-506 (PMC4227105; doi:10.1186/1471-2407-14-506)
Supplement: Additional file 6 — Ingenuity pathway analysis results for the 218 genes hypermethylated across grade II, III and IV tumor samples. Table S4. shows the top 5 molecular and cellular functions alongside the number of genes falling within each category and the p-value range. Gene symbols are given for genes that fall within any of these 5 categories. Gene symbols in bold indicate genes that have previously been associated with cancer. Table S5. illustrates the top 3 gene networks of the 218 hypermethylated genes and the genes present within each of these networks. [file 1471-2407-14-506-S6.docx]

**Table S4 - Ingenuity analysis of the 218 genes demonstrating the top 5 molecular and cellular functions**

| **Molecular and cellular function** | **No. of**  **Genes** | **P-value**  **range** | **Complete gene list for all 5 molecular and cellular functions**  **(Genes also associated with cancer by Ingenuity in bold)** |
| --- | --- | --- | --- |
| Cell morphology | 52/219  (23.7%) | 8.90E-08 –  9.86E-03 | *ACRBP,* ***ACTG1****,* ***ADAM12****,* ***ADRA1A****, ALS2CL,* ***ANXA2****,* ***APBA2****, ATP5G2, BCAT1,* ***BMP4****, BNC1,* ***CACNA1C****, CACNG8, CBLN3, CFLAR,* ***CFTR****, CNGA3,* ***CNTN2****, COL11A2, CPLX1,* ***CRIP1****,* ***CYBA****,* ***CYP11A1****,* ***DKK1****, DLGAP3,* ***EFEMP1****,* ***ERBB2****, FLOT1,* ***GDNF****, GFI1, GNAL,* ***GNMT****, GRASP, GRID2IP, GRIN1, GSX2, HNRNPF,* ***HSD11B2****, IDUA,* ***IER3****, KCNIP3, KIF5C, LHX5,* ***LZTS2****, M1AP, MAGI2,* ***MAL****, MAPT, MBP, mir-375, MYRIP, NBL1,* ***NEFM****,* ***NEUROD1****, NFKBIZ,* ***NID2****, NPM2, NR4A1, NRG2,* ***NRXN1****,* ***NTN1****,* ***NUAK1****,* ***ONECUT1****, ONECUT2, PAX6, PCDHGA4, PDE4A, PDLIM4,* ***PDPN****, PICK1,* ***PROM1****, PSD, PTH1R,* ***PXDN****,* ***PYCARD****,* ***QPCT****, RAB27B, RASGRP2,* ***RBP1****, RGMA, RHOD, SCGB3A1,* ***SERPINB1****, SHANK1, SHH, SIX3,* ***SOCS3****, SOST, SPEG, SSH3, SST, SSTR1, TBR1, TET1, TET2,* ***THRB****,* ***TNXB****, TOM1L1, TXNRD1, UNC13A, VAX2,* ***WNK2****, ZMYND10* |
| Cellular movement | 45/219  (20.5%) | 5.05E-07 –  9.86E-03 |  |
| Cellular development | 47/219  (21.5 %) | 9.41E-07 –  9.32E-03 |  |
| Cellular growth and proliferation | 65/219  (29.7%) | 3.74E-06 –  9.32E-03 |  |
| Cellular assembly and organization | 50/219  (22.8 %) | 4.14E-06 –  9.86E-03 |  |

**Table S5 - Ingenuity analysis of the 218 genes demonstrating the top 3 gene networks**

| **Network** | **Molecules in network** | **Focus molecules** | **Top functions** |
| --- | --- | --- | --- |
| **1** | ANXA2, CACNA1C, CELF4, CYBA, DKK1, FAM132A, GDNF, HSD11B2, ME1, mir-375, MYRIP, NRG2, NTN1, PAX6, PDE4A, PDPN, RAB27B, SOST, SST, TBR1, TCEA3, TET2, THRB, TOM1L1, VAX2, VRK2 | 26/219 (11.9%) | cell morphology, molecular transport, small molecule biochemistry |
| **2** | ACTA1, ACTG1, ATP5G2, BNC1, CFTR, CPLX1, CRIP1, ERBB2, GNMT, HNRNPF, KIF5C, MBP, ONECUT2, PDLIM4, PICK1, PLA2R1, RASGRP2, RHOD, SERPINB1, SIX3 | 20/219 (9.1%) | tissue development, cell morphology, cell-to-cell signalling and interaction |
| **3** | ACSL1, APBA2, CLSTN1, CMYA5, DDN, DLGAP3, FLOT1, GRASP, GRIN1, GSX2, MAGI2, MAPT, MYL12A, NRXN1, NUAK1, PROM1, RBP1, RBP7, SCGB3A1, SHANK1, | 20/219 (9.1%) | cell-to-cell signalling and interaction, cellular assembly and organization, nervous system development and function |
