# Supplementary material for: Stability of the CpG island methylator phenotype during glioma progression and identification of methylated loci in secondary glioblastomas
Source: BMC Cancer. 2014 Jul 10;14:506. doi: 10.1186/1471-2407-14-506 (PMC4227105; doi:10.1186/1471-2407-14-506)
Supplement: Additional file 8: Figure S3 — Venn diagram to illustrate the cross-over between hypermethylated genes within the grade IV pGBM and grade IV sGBM specific lists in addition to the original list of universally methylated genes across grade II, III and IV secondary glioma samples. [file 1471-2407-14-506-S8.pptx]

## Slide 1
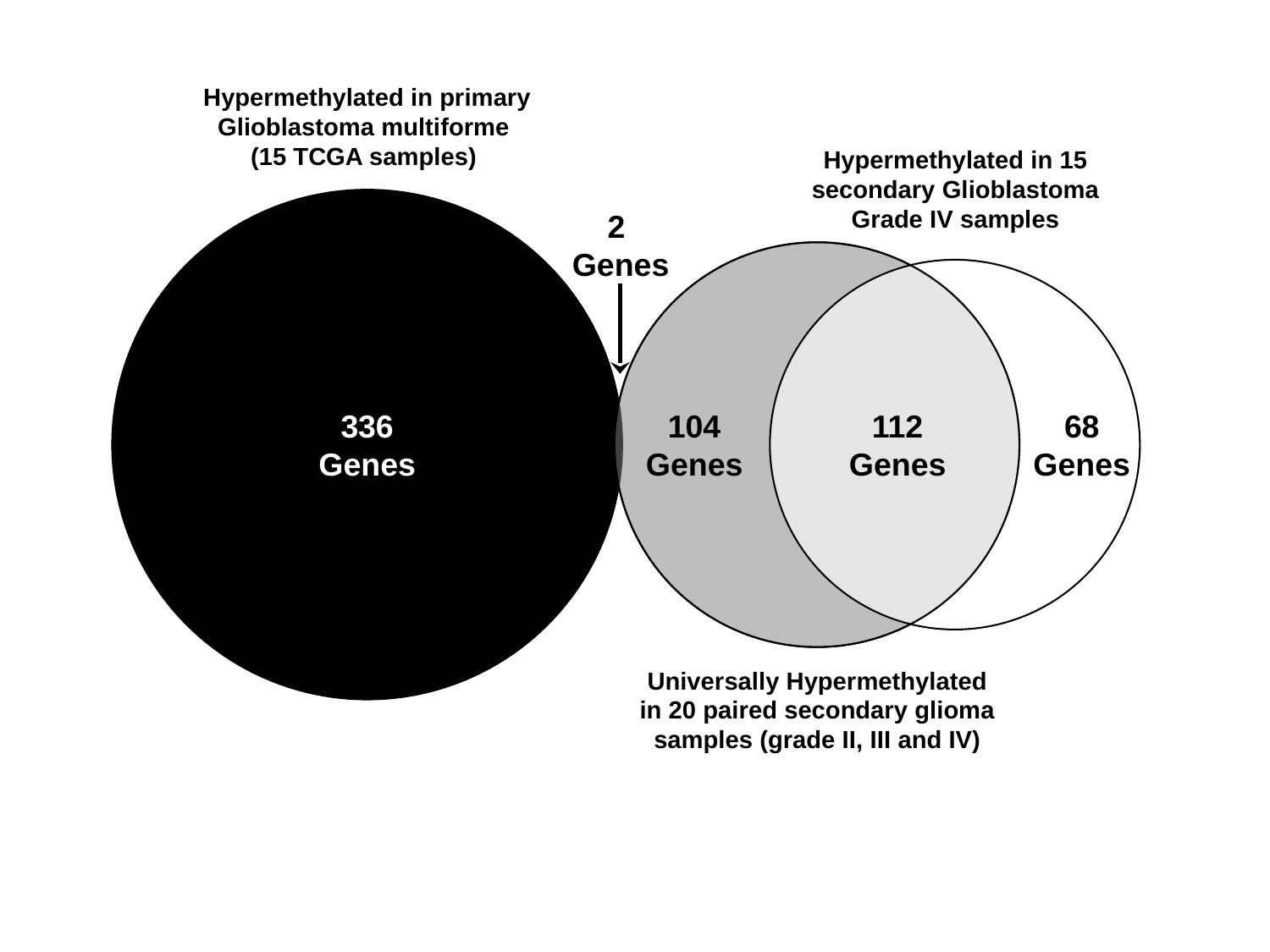

Hypermethylated in primary Glioblastoma multiforme
(15 TCGA samples)
Hypermethylated in 15 secondary Glioblastoma Grade IV samples
2 Genes
336 Genes
104 Genes
112 Genes
68 Genes
Universally Hypermethylated in 20 paired secondary glioma samples (grade II, III and IV)
